# Mechanical Deformation Mechanisms and Properties of Prion Fibrils Probed by Atomistic Simulations

**DOI:** 10.1186/s11671-017-1966-3

**Published:** 2017-03-29

**Authors:** Bumjoon Choi, Taehee Kim, Eue Soo Ahn, Sang Woo Lee, Kilho Eom

**Affiliations:** 1 Department of Biomedical Engineering, Yonsei University, Wonju, 26493 Republic of Korea; 2 College of Sport Science, Sungkyunkwan University (SKKU), Suwon, 16419 Republic of Korea; 3 Biomechanics Laboratory, College of Sport Science, Sungkyunkwan University (SKKU), Suwon, 16419 Republic of Korea

**Keywords:** Prion fibril, Mechanical deformation mechanism, Fracture property, Atomistic simulation

## Abstract

Prion fibrils, which are a hallmark for neurodegenerative diseases, have recently been found to exhibit the structural diversity that governs disease pathology. Despite our recent finding concerning the role of the disease-specific structure of prion fibrils in determining their elastic properties, the mechanical deformation mechanisms and fracture properties of prion fibrils depending on their structures have not been fully characterized. In this work, we have studied the tensile deformation mechanisms of prion and non-prion amyloid fibrils by using steered molecular dynamics simulations. Our simulation results show that the elastic modulus of prion fibril, which is formed based on left-handed β-helical structure, is larger than that of non-prion fibril constructed based on right-handed β-helix. However, the mechanical toughness of prion fibril is found to be less than that of non-prion fibril, which indicates that infectious prion fibril is more fragile than non-infectious (non-prion) fibril. Our study sheds light on the role of the helical structure of amyloid fibrils, which is related to prion infectivity, in determining their mechanical deformation mechanisms and properties.

## Background

Amyloid fibrils formed by protein aggregation have recently received significant attention due to their role in pathogenesis of neurodegenerative diseases [1] such as Alzheimer’s disease [2], Parkinson’s disease [3], and Creutzfeldt-Jakob disease [4]. These amyloid fibrils exhibit the structural feature in that they are formed as a one-dimensional ordered structure [5] with its thickness of ~1 nm and the length of >1 μm. These fibrils are quite stable in a physiological condition such that amyloid fibrils are not easily dissolved in a physiological condition. The stability of amyloid fibrils is attributed to their structural characteristics in that they are formed based on the β-sheet structure, which is a mechanically strong protein building block [6].

In recent years, amyloid fibrils have been highlighted for their remarkable mechanical properties [7, 8], which are comparable to those of mechanically strong protein materials such as spider silk [9, 10]. Specifically, recent studies [11–16] report that, based on atomic force microscopy (AFM) experiments, the elastic modulus of amyloid fibrils is measured in the order of 1 GPa. In addition, computational simulations based on atomistic or coarse-grained models provide that the elastic modulus of amyloid fibrils is evaluated in the order of 1 to 10 GPa [8, 17–22]. Here, we note that the larger value of elastic modulus measured by atomistic simulation is due to the loading rate used in simulation being a few orders of magnitude larger than the rate considered in AFM experiments. As the pulling speed increases, so does the elastic modulus of amyloid fibril [23]. Moreover, the fracture toughness of amyloid fibrils with their length scale of ~3 nm is found to be ~30 kcal mol^−1^ nm^−3^ [23], which is comparable to that of spider silk protein crystal with its length scale of ~2 nm [10]. The remarkable mechanical properties of amyloid fibrils have recently been found to be related to their biological function [7]. For instance, the mechanical disruption of cell membrane due to amyloid fibril [24] is ascribed to the elastic modulus of cell membrane being in the order of 100 kPa [25], which is about three orders of magnitude smaller than the elastic modulus of amyloid fibril [26]. This indicates the important role of the mechanical properties of amyloid fibrils in their biological functions. Furthermore, a recent study by Weissman and coworkers [27] has shown a correlation between the brittleness of prion fibrils and prion infectivity. Our recent study [19] reports that the size-dependent elastic properties of prion fibrils provide an insight into their critical size of infectious prion fibrils. In addition, a recent study by Choi et al. [28] reports the mechanical and structural characteristics of prion fibrils under different pH conditions with using elastic network model (ENM)-based normal mode analysis.

The mechanical properties of amyloid fibrils have been probed by a force spectroscopy based on optical tweezer or AFM, which allows for characterizing the mechanical behavior of protein materials in response to a force [29]. For example, an optical tweezer-based force spectroscopy has been employed to study the mechanical behavior of human prion protein PrP (90–231) [30] and yeast prion protein Sup35 [31]. AFM-based force spectroscopy has been used to characterize the radial compression behavior of prion fibril [32]. In addition, a force spectroscopy based on optical tweezer has been utilized to characterize the mechanical unfolding and refolding of a single prion protein PrP in order to gain insight into prion misfolding related to the formation of amyloid oligomers that serve as a nucleation seed [33]. Though force spectroscopy-based experiments are able to characterize the mechanical properties of amyloid fibrils, they are restrictive for understanding the structure-property relationship of amyloid fibrils because they are unable to provide the structural deformation mechanisms of amyloid fibrils at atomic resolution. However, an atomistic simulation can visualize the structural deformation of protein material in response to a force [34–37], which suggests that atomistic simulation provides the detailed insight into the deformation mechanisms of protein materials. For instance, our previous study [23] has reported the mechanical deformation mechanism of human islet amyloid polypeptide (hIAPP) fibrils by using steered molecular dynamics (SMD) simulations. Recent studies by Na and colleagues [38, 39] provide the bond rupture mechanisms of amyloid fibrils under a force based on SMD simulations. Buehler and coworkers have utilized SMD simulations to understand the mechanical deformation mechanisms and properties of protein materials such as spider silk proteins [10, 40] and amyloid fibrils [21, 22, 41]. Furthermore, in our previous work [18], molecular dynamics (MD) simulation was used to investigate the role of steric zipper pattern in the elastic properties of hIAPP fibrils. We have also considered atomistic simulations to study the unfolding mechanism of a single prion protein under a force [42]. These examples underlie the ability of atomistic simulations to provide insight into the structural deformation mechanisms of protein materials in response to a force.

Despite recent studies reporting the mechanical properties of amyloid fibrils, the mechanical deformation mechanisms and fracture behaviors of prion amyloid fibrils have not been fully characterized. Though our recent study [19] provides the elastic properties of prion and non-prion amyloid fibrils, the mechanical deformation characteristics and fracture properties of prion and non-prion fibrils have not been fully understood. Here, we note that ENM [43–45] used in our recent study [19] is unable to provide any insight into the mechanical deformation mechanisms of amyloid fibrils, since ENM is applicable for analysis of elastic properties for protein materials undergoing small deformation. Here, we study the mechanical deformation mechanisms and fracture properties of prion and non-prion amyloid fibrils using SMD simulations. Our simulations are aimed towards unveiling how the helical structure of (prion) amyloid fibrils determines their mechanical deformation mechanisms and properties. We found that the helical structure determines not only the elastic properties of (prion) amyloid fibrils but also their deformation mechanisms such as the failure pathways of prion fibrils. Specifically, our simulation results show that infectious prion fibrils can be more easily fragmented (or ruptured) than (non-infectious) non-prion fibril, and that the fracture toughness of (prion) amyloid fibrils is encoded in their helical structures. Our study provides insight into a design rule showing how the fracture toughness of (prion) helical amyloid fibrils are determined.

## Methods

**Fig. 1:**
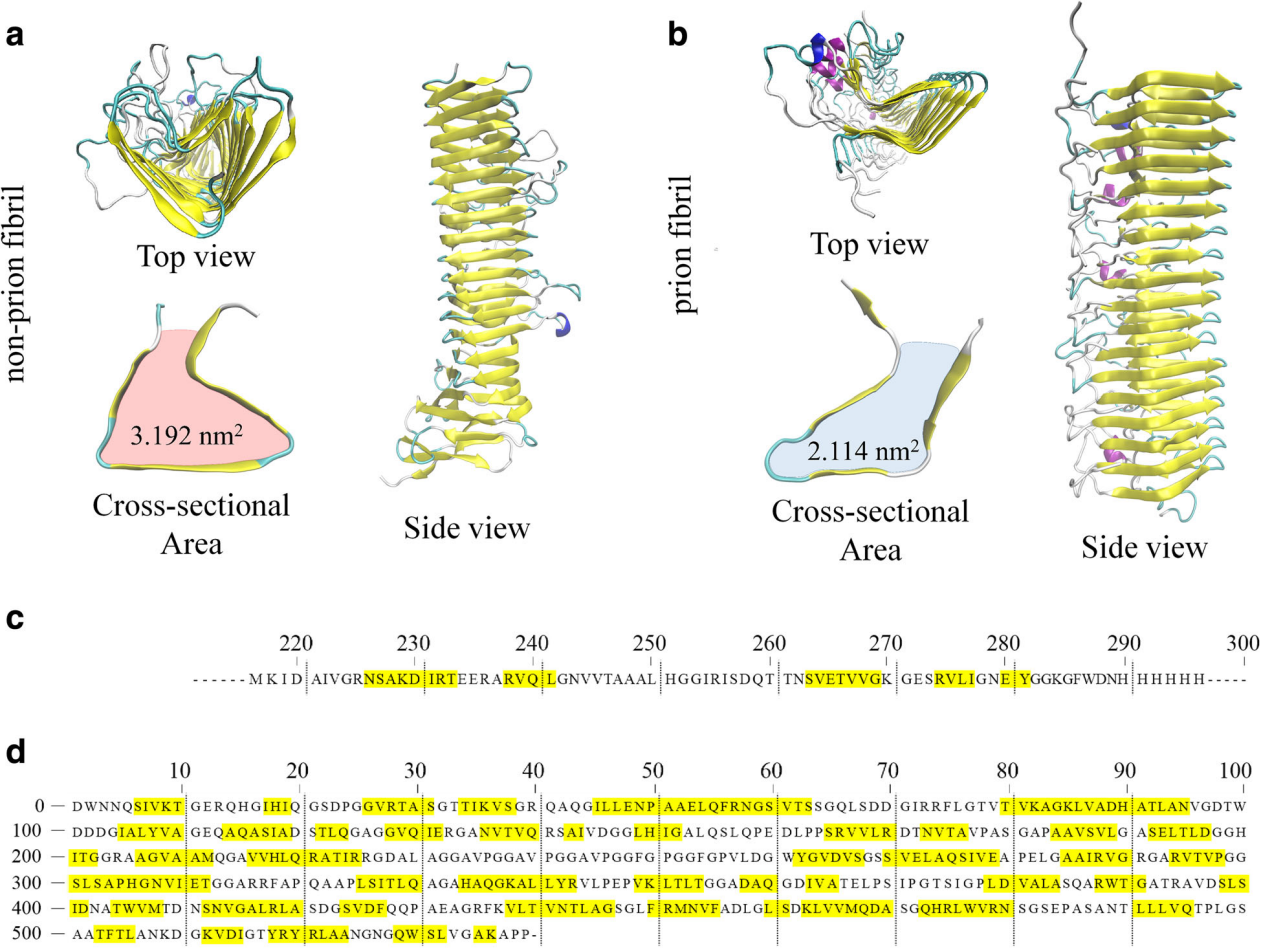
**a** Molecular structure of non-prion p69 pertactin fibril. **b** Atomistic structure of HET-s prion fibril. **c** Sequence of the segment of HET-s prion fibril. Here, HET-s prion fibril considered in this work is made of nine repeated segments. **d** Sequence of p69 pertactin fibril. It should be noted that the consecutive *sequences highlighted in yellow* form a β-strand

We consider HET-s prion fibril and (non-infectious) p69 pertactin fibril, both of which are made of β-helical structure. In particular, the (HET-s) prion fibril is made of left-handed β-helix, while the non-prion (p69 pertactin) fibril is constructed based on right-handed β-helical structure. The molecular structures of prion and non-prion fibrils are deposited in protein data bank (pdb) with pdb code of 2RNM (for prion fibril) and 1DAB (for non-prion fibril), respectively. Here, we note that the length of prion and non-prion fibrils is measured as 8.2 and 8.4 nm, respectively. These molecular structures are presented in Fig. 1.

To obtain the equilibrium structures of these fibrils, we utilized NAMD package [46] with CHARMM27 force field [47]. Here, the fibril was solvated using explicit water molecules modeled as TIP3P. Here, the box of explicit water molecules is constructed in such a way that the distance between the outer surface of water box and the fibril is set to be 2 nm. Before, equilibration, we performed energy minimization process using conjugate gradient method with 10,000 steps. The cut-off and switching distance for non-bonded interactions is set to be 1 and 1.2 nm, respectively. Then, the fibril structure is equilibrated for 50 ns under NPT ensemble at 310 K and 1 atm with time step of 2 fs based on SHAKE algorithm. For NPT ensemble-based molecular dynamics simulations, the particle mesh Ewald (PME) is used with PME size of 0.9 nm. The equilibrium dynamics simulation based on NPT ensemble was conducted based on Langevin thermostat and Nose-Hoover barostat in order to make the temperature and pressure be constant. The equilibrium dynamics trajectories and energy values are recorded for every 2 and 0.2 ps, respectively.

To pull the amyloid fibril along the fibril axis, we considered SMD simulations that give rise to the mechanical deformation of protein materials in silico. In order to extend the amyloid fibril along the fibril axis, we fix the bottom three layers of the fibril, while a spring mimicking a force probe is attached to the center of mass for top three layers of the fibril. Then, the fibril is pulled along the fibril axis by moving a spring (whose force constant is given by 12 kcal mol^-1^ Å^-1^) with a constant velocity in a range of 0.001 to 0.05 Å/ps. Here, SMD simulations were performed based on NVT ensemble, and these simulations was conducted until the fibril structure is entirely fractured. The SMD trajectories are recorded for every 1 ps.

## Results and Discussion

In this work, we consider HET-s prion fibril [48] and p69 pertactin fibril (that is a non-prion fibril) [49], whose structures are available in protein data bank (for more detail, see “Methods” section). It should be noted that the mechanical properties of protein materials are determined from their molecular structure (i.e., morphology) rather than their sequence. For instance, the native topology of a single protein molecule determines the mechanical unfolding mechanism of a protein molecule [50, 51]. Specifically, the mechanical strength of a protein molecule is determined from the network of hydrogen bonds that stabilize a protein structure [52]. In addition, the bending property of amyloid fibrils is related to their cross-sectional shape as predicted by elasticity theory [13, 53]. As shown in Fig. 1, despite the sequence difference between HET-s fibril and p69 pertactin fibril, these two fibrils exhibit similar cross-sectional shape such that three β-strands form a triangular cross-sectional shape for these fibrils. It is shown that though the cross-sectional area of prion fibril is similar to that of non-prion fibril, the solvent accessible surface area (SASA) of prion fibril is different from that of non-prion fibril. In particular, the SASA of prion fibril is measured as ~4 × 10^2^ nm^2^, while the SASA of non-prion fibril is estimated as ~2.5 × 10^2^ nm^2^. This indicates that non-prion fibril is more hydrophobic than prion fibril. As an increase in the number of water molecules surrounding the amyloid fibrils reduces their mechanical stability and properties [54], the non-prion fibril is anticipated to be mechanically stronger than prion fibril. In addition, the HET-s prion fibril is formed based on stacked β-sheets with left-handed helical pattern, while non-prion p69 pertactin fibril is constructed based on right-handed β-helix. Though our previous study [19] reports the role of helical pattern on the elastic properties of prion and non-prion fibrils, the effect of the helical pattern on the mechanical deformation mechanisms and fracture properties of prion and non-prion fibrils has not been characterized; this study is aimed to provide insight into this effect on the mechanical deformation mechanisms and fracture properties of prion fibrils.

**Fig. 2:**
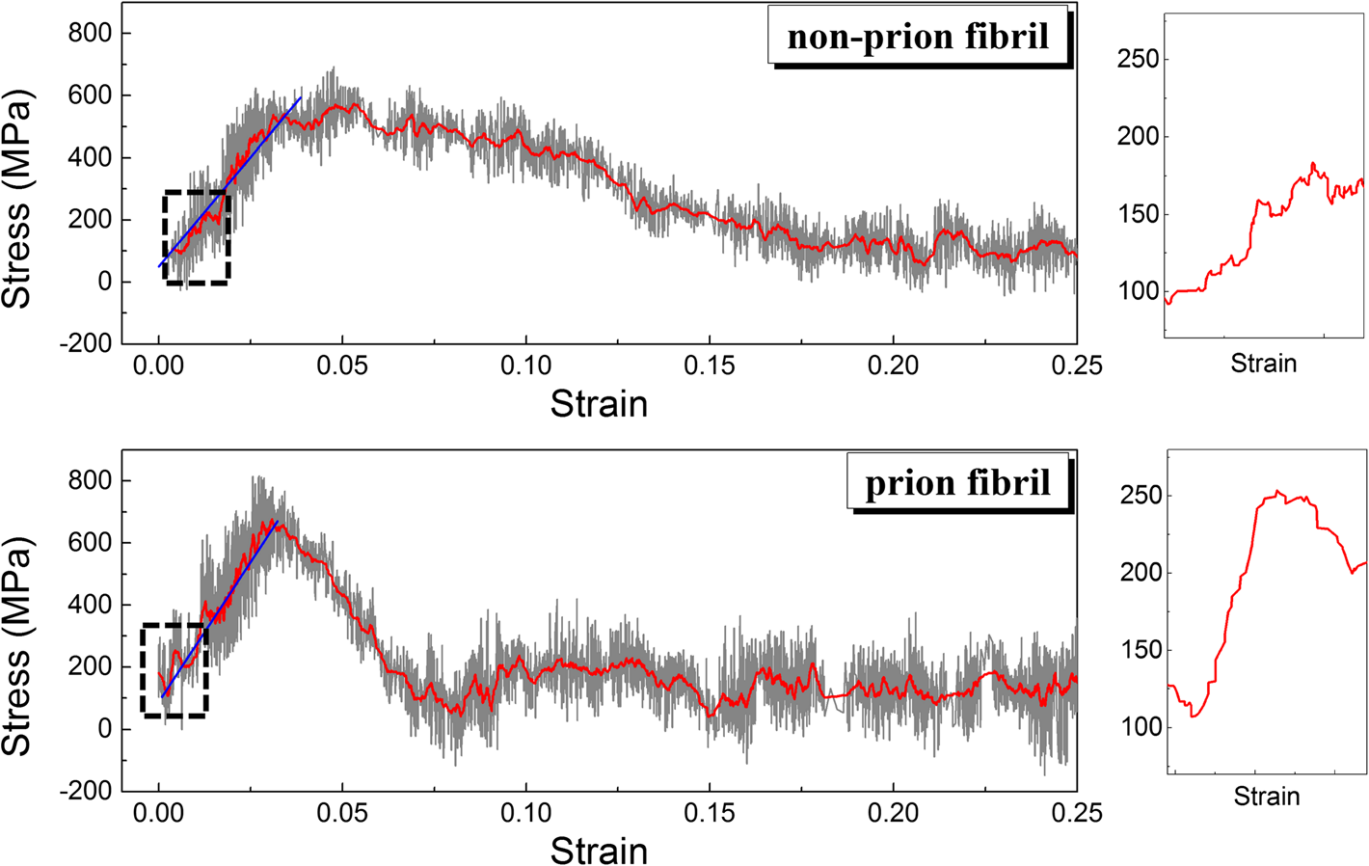
Stress-strain curves for non-prion and prion fibrils, when these fibrils are pulled along the fibril axis. The *black dashed* region of the stress-strain curve is shown in the *right panel*

**Fig. 3:**
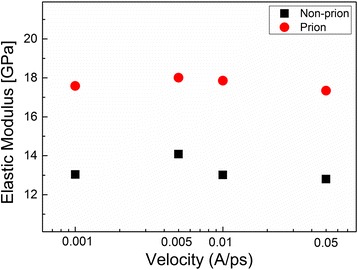
Elastic modulus of prion and non-prion fibrils as a function of pulling speed

As our previous study [19] reports that the axial elastic modulus of prion fibril is larger than that of non-prion fibril, we study the tensile deformation behaviors of prion and non-prion fibrils using SMD simulations. Figure 2 shows the stress-strain curves of prion and non-prion fibrils, respectively. It is shown that for a non-prion fibril, the first peak of stress is measured as 200 MPa at a strain of ~0.55%. On the other hand, the first peak of stress for prion fibril is estimated as 150 MPa at a strain of ~0.15%. This suggests that the prion fibril begins to be ruptured at the stress of ~150 MPa, which is smaller than the stress (i.e., ~200 MPa) at which non-prion fibril starts to be ruptured. This indicates that the prion fibril exhibits the lower resilience than non-prion fibril, which is consistent with a recent finding [27] that infectious prion fibril is more likely to be fragile than non-infectious fibrils. However, the maximum stress (referred to as strength), at which fibril is significantly ruptured, is measured as ~620 MPa for both prion and non-prion fibrils (Fig. 3). Here, we note that though the strength of non-prion fibril is comparable to that of prion fibril, the strain at the stress of ~620 MPa is measured as ~3.7% for non-prion fibril, while it is estimated as ~2.5% for prion fibril (Fig. 2). We note that difference between the values of strains (at the stress of ~620 MPa) for prion and non-prion fibrils may be attributed to the deformation mechanisms of these fibrils (see below). In addition, we measured the elastic modulus of prion and non-prion fibrils, respectively, which were pulled along the fibril axis. Here, the elastic modulus of the fibril is measured as a slope in the linear region of the force curve based on Hooke’s law such as *E* = ∂*σ*/∂*ε*, where *E*, *σ*, and *ε* are the elastic modulus, stress, and strain, respectively (see Fig. 2). The elastic modulus of non-prion and prion fibrils is measured as ~13 and ~18 GPa, respectively, when these fibrils were extended with a pulling speed in a range of 0.001 to 0.05 Å/ps (Fig. 3). This indicates that the prion fibril exhibits the higher axial elastic modulus than non-prion fibril, which is consistent with our previous finding [19]. In addition, the (tensile) elastic modulus of prion and non-prion fibrils is almost independent of pulling speed for its range of 0.001 to 0.05 Å/ps. The elastic moduli of prion and non-prion fibrils measured from our SMD simulations are an order of magnitude larger than those estimated from ENM simulations reported in our previous work [19]. This is attributed to the rate effect considered in SMD simulations, while ENM simulations ignore the rate effect. The values of elastic modulus of both prion and non-prion fibrils are comparable to the elastic modulus of Aβ fibrils (i.e., ~15 GPa) [55] measured from SMD simulations. It should be noted that though both amyloid fibril and spider silk crystal are formed based on stacking of β-sheets, the elastic moduli of amyloid fibrils studied in this work and refs. [8, 21, 38] are smaller than the elastic modulus of spider silk crystal (i.e., ~60 GPa) [55]. The larger value of elastic modulus for spider silk crystal than that for amyloid fibril is ascribed to the loading mechanism in that the spider silk crystal bears a loading (force) whose direction is perpendicular to the fibril axis of spider silk crystal, while the amyloid fibril exerts a force along the fibril axis [55]. This indicates the important role of loading direction in the elastic properties of protein fibrils including amyloid fibrils. The effect of loading directions in the mechanical deformation mechanisms and properties of amyloid fibrils will be considered in our future study.

**Fig. 4:**
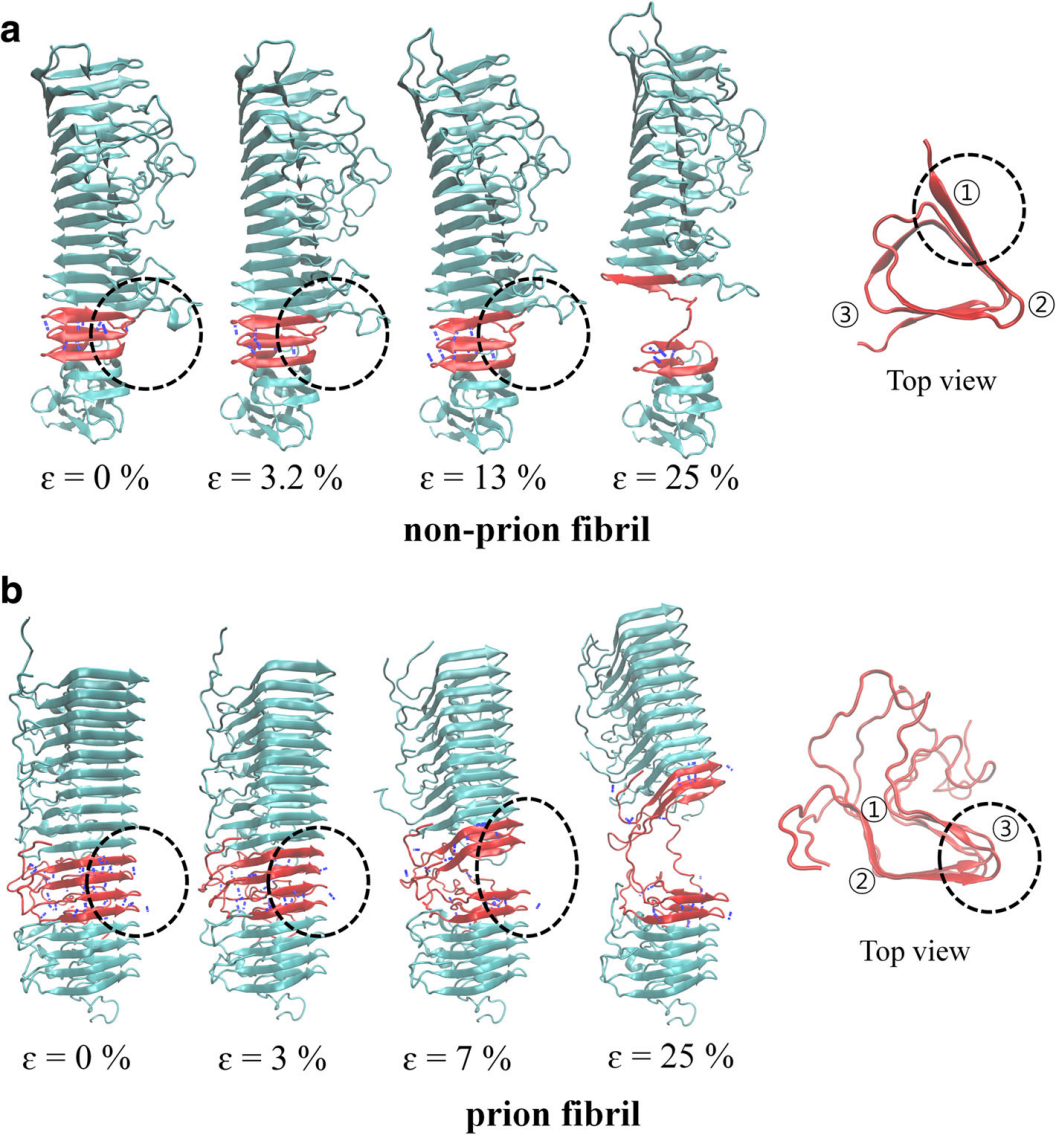
Deformation and failure pathways of **a** non-prion fibril and **b** prion fibril as a function of strain. The *black dashed circles* indicate the region at which the fracture of the fibril begins

To gain insight into the mechanical behaviors of prion and non-prion fibrils, we consider the deformation and failure pathway of these fibrils. In particular, Fig. 4 shows the atomistic structures of these fibrils a function of strain, when these fibrils were extended with a pulling speed of 0.005 Å/ps. It is found that when both prion and non-prion fibrils are extended along the fibril axis, β-helical layers close to the bottom layer of these fibrils begin to be ruptured. Moreover, for both prion and non-prion fibrils, a hydrogen bond at the vertex of triangle-shaped layer is initially broken (Fig. 4). In addition, the deformation and failure pathways of these fibrils are independent of extension rate in a range of 0.001 to 0.05 Å/ps (not shown).

**Fig. 5:**
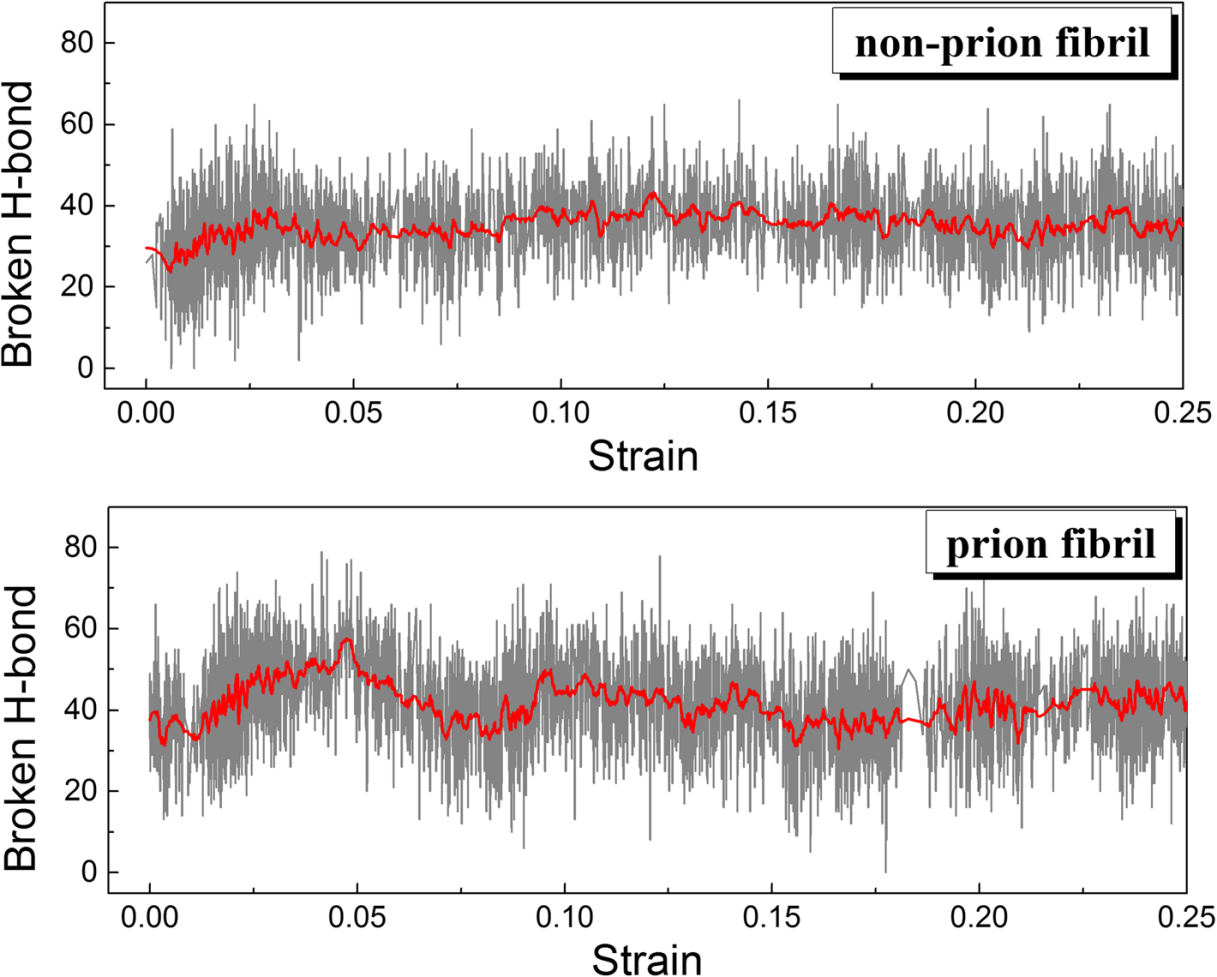
The number of ruptured hydrogen bonds for prion and non-prion fibrils during their deformation process

To understand how the fracture behavior of amyloid fibrils is determined, we investigate the hydrogen bond rupture mechanisms of prion and non-prion fibrils during their deformation processes. Figure 5 shows the number of hydrogen bonds that are ruptured during the deformation pathways of prion and non-prion fibrils. Here, the number of ruptured hydrogen bonds (*N*_*r*_) is calculated such as *N*_*r*_(*t*) = *N*_*HB*_(0)–*N*_*HB*_(*t*), where *N*_*HB*_(*t*) is the number of hydrogen bonds (sustaining the fibril structure) at time *t*. It is found that the number of ruptured hydrogen bonds increases with respect to strain until the fibril is significantly fractured. For non-prion fibril, as the strain increases, the number of ruptured hydrogen bonds is increasing even up to ~40, before the strain reaches ~3.7%. However, for prion fibril, as the strain increases, so does the number of ruptured hydrogen bonds even up to ~60 until the strain of ~5%. We note that the number of fractured hydrogen bonds for prion fibril is measured as ~40 at the strain of ~2.5%. Our results suggest that the mechanical strength of amyloid fibril is closely related to the number of the ruptured hydrogen bonds of the fibril. In particular, the number of fractured hydrogen bonds for prion fibril is larger than that for non-prion fibril, which indicates that non-prion fibril is mechanically stronger than prion fibril. In addition, it is shown that for a prion fibril, the number of ruptured hydrogen bonds decreases for the strain in a range of 5 to 7%, which implies that the neighboring two β-helical layers of a prion fibril are not completely separated at strain of 7% (e.g., see Fig. 4). Specifically, the neighboring layers of a prion fibril are somewhat separated, but these layers are connected by two loops at the strain of 7%. Here, we note that the fracture behaviors and properties of amyloid fibrils are determined from hydrogen bonding interaction (between the β-helical layers of amyloid fibril) rather than other intermolecular interactions such as electrostatic interaction. Though electrostatic interaction plays a role in self-assembly process to form an amyloid fibril [56, 57], the electrostatic interaction energies of both prion and non-prion fibrils do not change during the failure (fracture) process of the fibrils (not shown), which suggests that electrostatic interaction energy does not play any role in determining the mechanical strength of amyloid fibrils.

**Fig. 6:**
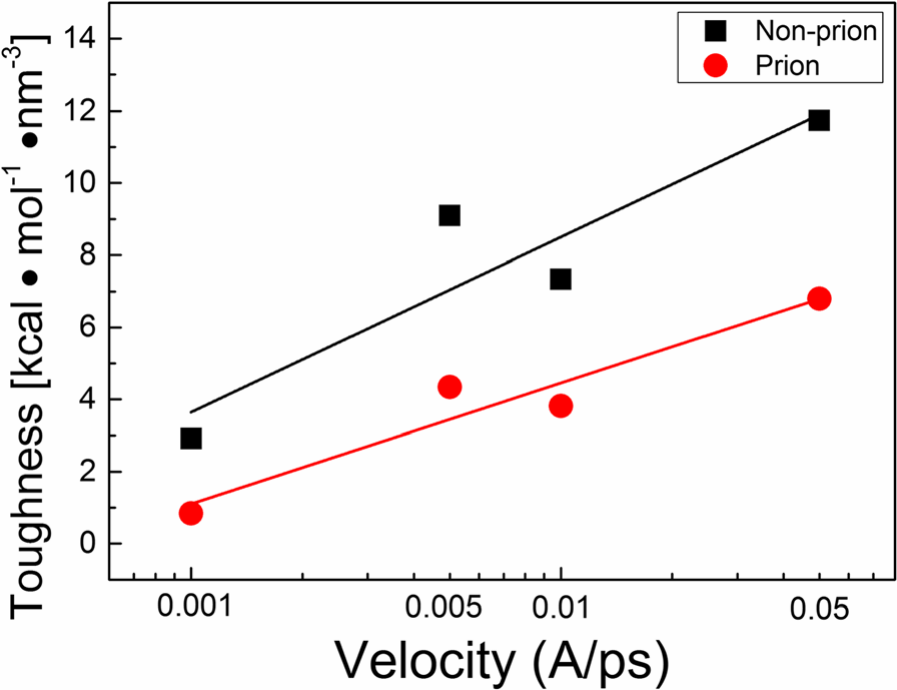
Mechanical toughness of non-prion and prion fibrils as a function of pulling speed

To characterize the fracture properties of prion and non-prion fibrils, we measured the mechanical toughness (*E*_*T*_) of prion and non-prion fibrils, which is defined as

1 $$ {E}_T={\displaystyle {\int}_0^{\varepsilon_{\mathrm{o}}}\sigma \left(\varepsilon \right)} d\varepsilon $$

where *σ*(*ε*) represents the stress of fibril as a function of strain (*ε*) and *ε*_0_ is the strain at which the neighboring layers of the fibril is completely separated. It is shown that the mechanical toughness of non-prion fibril is larger than that of prion fibril (Fig. 6), which agrees with the mechanical strength of non-prion fibril being higher than that of prion fibril (e.g., Fig. 3). This observation is consistent with a recent finding [27] that the infectious prion fibril is more fragile than non-infectious fibril. In addition, the toughness of prion and non-prion fibrils (with their length of ~8 nm) is measured as 3 and 7 kcal mol^−1^ nm^−3^, respectively. The toughness of prion and non-prion fibrils is larger than that (i.e., ~2 kcal mol^−1^ nm^−3^) of human islet amyloid polypeptide (hIAPP) fibril with its length of 8 nm [38]. This suggests that amyloid fibril made of β-helical structure is mechanically tougher than the fibril composed of stacked β-sheets, which is consistent with the recent finding of Solar and Buehler [21]. Furthermore, we note that the toughness of amyloid fibril is dependent on the loading mode (direction), that is, deformation mechanism. In particular, when the hIAPP amyloid fibril with its length of ~8 nm undergoes the bending deformation, the toughness of the fibril is evaluated as ~10 kcal mol^−1^ nm^−3^ [23], which is larger than the toughness of the fibril when it is pulled along the fibril axis. Furthermore, the toughness of both prion and non-prion fibrils is dependent on the pulling speed in such a way that the toughness is linearly proportional to the logarithm value of pulling speed (Fig. 6). This can be elucidated based on Bell’s theory [58], which suggests that the rupture force (*F*_*r*_) and mechanical strength (*σ*_*s*_) of amyloid fibril are linearly proportional to the logarithm value of pulling speed (*v*) such as *F*_*r*_ = *F*_0_ln*v* and *σ*_*s*_ = *σ*_0_ln*v* [23]. As the mechanical behavior of amyloid fibrils can be dictated by linear elasticity (as shown in Fig. 2), the mechanical toughness is shown to be linearly proportional to the logarithm value of pulling speed such as *E*_*T*_ ∝ *σ*_*s*_ *ε*_0_ ∝ *σ*_0_ *ε*_0_ln*v*.

## Conclusions

In this work, we have studied the mechanical (tensile) deformation mechanisms and fracture properties of both prion and non-prion fibrils using SMD simulations. It is found that the axial elastic modulus of prion fibril is larger than that of non-prion fibril, whereas the mechanical toughness and strength of prion fibril are smaller than those of non-prion fibril. This result is consistent with recent finding [27], which suggests that infectious prion fibrils are more fragile than non-infectious fibrils. It is shown that the helical structure of prion amyloid fibrils plays a role in determining the mechanical deformation mechanisms and properties of these fibrils. In particular, the fracture behavior and property of the fibril are determined from the rupture mechanisms of hydrogen bonds that stabilize interactions between the neighboring helical layers of the fibril. Our study provides insight into how the β-helical structure of prion fibrils governs their mechanical (tensile) deformation mechanisms and properties.
